# Trends and Disparities in Colonic Diverticular Disease Hospitalizations in Patients With Morbid Obesity: A Decade-Long Joinpoint Analysis

**DOI:** 10.7759/cureus.36843

**Published:** 2023-03-29

**Authors:** Pius E Ojemolon, Hafeez Shaka, Robert Kwei-Nsoro, Philip Kanemo, Mihir Shah, Abdulrahman I Abusalim, Bashar Attar

**Affiliations:** 1 Internal Medicine, John H. Stroger, Jr. Hospital of Cook County, Chicago, USA; 2 Internal Medicine, Rapides Regional Medical Center, Alexandria, USA; 3 Internal Medicine, Englewood Hospital, New Jersey, USA; 4 Gastroenterology and Hepatology, Rush University Medical Center, Chicago, USA; 5 Gastroenterology and Hepatology, John H. Stroger, Jr. Hospital of Cook County, Chicago, USA

**Keywords:** joinpoint analysis, complicated diverticular disease, diverticular disease, morbid obesity, trends

## Abstract

Objective

We aimed to describe epidemiologic trends in outcomes of colonic diverticular disease (CDD) hospitalizations in morbidly obese patients.

Methods

We searched the United States National Inpatient Sample databases from 2010 through 2019, obtained the incidence rate of morbid obesity (MO) among CDD hospitalizations, and used Joinpoint analysis to obtain trends in these rates adjusted for age and sex. Hospitalizations involving patients less than 18 years of age were excluded. Trends in mortality rate, mean length of hospital stay (LOS), and mean total hospital charge were analyzed. Multivariate regression analysis was used to obtain trends in adjusted mortality, mean LOS, and mean total hospital charge.

Results

We found an average annual percent change of 7.5% (CI = 5.5-9.4%, p < 0.01) in the adjusted incidence of MO among hospitalizations for CDD over the study period. We noted a 7.2% decline in mortality (p = 0.011) and a 0.1 days reduction in adjusted LOS (p < 0.001) over the study period. Hospitalizations among the middle-aged and elderly had adjusted odds ratios of 7.18 (95% CI = 2.2-23.3, p = 0.001) and 24.8 (95% CI = 7.9-77.9, p < 0.001), respectively, for mortality compared to those in young adults. The mean LOS was 0.29 days higher in females compared to males (p < 0.001).

Conclusion

The incidence of MO increased among CDD hospitalizations while mortality and mean LOS reduced over the study period. Outcomes were worse in older patients, with an increased mean LOS in females compared to males.

## Introduction

Colonic diverticulosis refers to the presence of diverticula that result from herniation of colonic mucosa and muscularis mucosa through the muscularis propria at sites of vascular penetration [1]. Diverticulosis is one of the most common gastrointestinal conditions and is often found incidentally on colonoscopy [2]. The prevalence of colonic diverticulosis is less than 10% in persons below 40 years of age but approaches 50-70% in patients aged over 80 years [3]. These diverticula are typically asymptomatic but 20-30% of individuals develop symptomatic diverticulosis, termed colonic diverticular disease (CDD) [3,4].

Diverticular disease can range in severity from symptomatic uncomplicated diverticular disease manifesting as bloating, mild abdominal pain, and changes in bowel habit to symptomatic disease with complications such as acute diverticulitis or diverticular hemorrhage, which often prompt hospitalization and may necessitate endoscopic procedures or surgical intervention [4]. Diverticular hemorrhage is the most common cause of lower gastrointestinal bleeding in the United States, accounting for 20-66% of cases [5]. However, fewer than 5% of patients with diverticulosis experience diverticular hemorrhage [4]. Similarly, only 1-4% of individuals with colonic diverticula will develop diverticulitis, with a recurrence risk of ~20% at five years [6].

Diverticular disease impacts markedly on patients' well-being and is one of the most economically significant diseases in gastroenterology. It accounts for almost two million ambulatory visits, over 370,000 emergency department visits, and over 200,000 hospital admissions, and contributes to over 4,600 deaths every year [1,7]. Healthcare expenditures spent on CDD in 2015 totaled US$5.5 billion, compared to the US$4.1 billion spent on colon cancer or the US$6.1 billion spent on other gastrointestinal cancers [7].

The incidence of obesity has risen in the US over the past half-century [8]. Obesity accounted for nearly US$173 billion in healthcare costs in 2019 [9]. Reports are conflicting on the relationship between obesity and asymptomatic diverticulosis [10]. Some studies have shown that there is no relationship [11], while others have demonstrated an increased incidence of asymptomatic diverticulosis in obese patients [12,13]. There is more clarity of evidence linking CDD to obesity. Obesity has been associated with progression from colonic diverticula to CDD, diverticular hemorrhage, diverticulitis, perforations, more severe or complicated disease, recurrent diverticulitis, and worse outcomes following surgical intervention [11,13-15]. However, it is unclear if morbid obesity (MO) is associated with worse outcomes in patients admitted for CDD. Thus, our aim was to elucidate epidemiologic trends and biodemographic disparities in the outcomes of hospitalizations for CDD in morbidly obese patients over the course of a decade.

## Materials and methods

Study design and data source

We carried out a retrospective longitudinal trends study involving CDD hospitalizations in morbidly obese patients in the United States. Data were sourced from the Nationwide Inpatient Sample (NIS) databases from 2010 to 2019. The NIS is a database of inpatient hospital stays derived from billing data submitted by hospitals to statewide data organizations across the US, covering more than 97% of the US population [16]. The dataset provided for a given calendar year approximates a 20% stratified sample of discharges from US community hospitals, excluding rehabilitation and long-term acute care hospitals. This dataset is weighted to obtain US national estimates [17]. Databases before 2016 were coded using the International Classification of Diseases, Ninth Revision, Clinical Modification (ICD-9-CM)/Procedure Coding System (PCS). Databases from 2016 were coded using the International Classification of Diseases, Tenth Revision, Clinical Modification (ICD-10-CM)/PCS. The 2015 NIS has both ICD-9 and 10 codes, hence requiring a combination of both codes to obtain the studied cohort in keeping with the Healthcare Cost and Utilization Project (HCUP) regulations [18]. In this study, we weighted the nine months of ICD-9 data in 2015 for the entire year. In the NIS, diagnoses are categorized into two separate groups: principal diagnosis and secondary diagnoses. A principal diagnosis was the International Classification of Diseases (ICD) code attributed as the reason for hospitalization. Secondary diagnoses were any ICD code discharge diagnosis other than the principal diagnosis. We obtained total yearly adult hospitalizations from the NIS databases.

Study population and variables

We searched the databases for hospitalizations for CDD and isolated those with MO using ICD codes. Hospitalizations involving patients less than 18 years were excluded. The NIS includes variables on patient demographics, including age, sex, race, and median household income (MHOI) for the patient’s zip code (income quartiles referred to patients as 1 - low income, 2 - middle income, 3 - upper middle income, and 4 - high income), and primary payer. It also contains hospital-specific variables, including bed size, teaching status, and location. We assessed the comorbidity burden using Sundararajan’s adaptation of the modified Deyo’s Charlson Comorbidity Index (CCI). This modification groups CCI into four groups in increasing risk for mortality. It has been adapted to population-based research. A score of >3 has about a 25% 10-year mortality, while a score of 2 or 1 has a 10% and 4% 10-year mortality, respectively. This cutoff point was chosen as a means of assessment of the increased risk of mortality [19]. Total hospital charge (THC) was adjusted for inflation using the Medical Expenditure Panel Survey index for hospital care, with 2019 as the reference point [20].

Outcome measures

Biodemographic trends of hospitalizations for CDD in patients with MO were highlighted. Specifically, we obtained the incidence of MO among CDD hospitalizations and expressed the rate as a percentage. We adjusted this rate for age and gender and used Joinpoint regression analysis to obtain trends in these adjusted rates.

We analyzed trends in inpatient mortality rate, mean length of hospital stay (LOS), and mean THC of both cohorts. We also highlighted disparities in outcomes of these hospitalizations stratified by sex (male and female), race (Whites, Blacks, and Hispanics), and MHOI quartile (low-income quartile (LIQ) and high-income quartile (HIQ)).

Statistical analysis

We used Stata® version 16 software (StataCorp LLC, College Station, TX) and Joinpoint Regression Program version 4.9.1.0 (National Cancer Institute, Bethesda, MD) for data analysis. We analyzed the weighted sample following HCUP regulations for utilizing the NIS database. Age was grouped as young adults (18-44 years), middle-aged adults (45-64 years), and elderly (65 years and above). The incidence of MO among CDD hospitalizations was calculated with the HCUP methodology for disease incidence [21]. We used multivariate regression analysis to calculate incidence rates adjusted for age, gender, and race using predictive margins. We then used Joinpoint regression analysis to obtain trends in these rates over the study duration using standard errors. Joinpoint regression analysis has previously been validated by the National Institute of Health (NIH) National Cancer Institute to model non-linear trends in cancer rates over a given period [22] and has been adopted in prior HCUP database research [23,24]. We used multivariate regression analysis to obtain trends in mortality, LOS, and THC adjusted for age categories, sex, and race. All p-values were two-sided. The threshold for statistical significance was set at 0.05.

Ethical considerations

This study did not require Cook County Health Institutional Review Board approval as the NIS database lacks patient and hospital-level identifiers.

Data availability statement

The NIS is a publicly available inpatient care database that contains data on more than seven million hospitalizations annually. Its large sample size makes it ideal for developing regional and national estimates and enables analyses of special populations, uncommon conditions, and rare treatments.

## Results

Incidence

Of the 305 million hospitalizations included in our study, more than 2.7 million were for CDD, with 142,110 of these patients being morbidly obese. The incidence rate of MO among hospitalizations with CDD increased from 3.4% in 2010 to 7.2% in 2019 (Table 1).

**Table 1:** Biodemographic data of hospitalizations with CDD and morbid obesity CCI: Charlson Comorbidity Index; CDD: colonic diverticular disease; MHOI: median household income national quartile for patient's ZIP code; MO: morbid obesity; SD: standard deviation from the mean.

Variable	2010	2011	2012	2013	2014	2015	2016	2017	2018	2019	P-value
Total hospitalizations, million, N = 305	31.4	31.5	30.7	30.0	29.8	30.2	30.2	30.4	30.3	30.2	
Total hospitalizations with CDD (N = 2,750,796)	292,639	312,468	302,090	292,515	290,150	273,395	235,035	245,305	250,520	256,680	
Total hospitalizations with CDD and MO (N = 142,110)	9,905	11,720	12,580	13,595	14,400	14,690	14,320	15,120	17,275	18,425	
MO incidence rate among hospitalizations with CDD, %	3.4	3.8	4.2	4.7	5.0	5.4	6.1	6.2	6.9	7.2	<0.001
Mean age ± SD, years	56.1 ± 14.8	55.7 ± 15.3	56.1 ± 14.6	56.2 ± 14.3	56.2 ± 15.1	56.4 ± 14.7	56.1 ± 14.8	56.4 ± 14.8	56.1 ± 14.9	56.9 ± 14.7	0.023
Age categories, %											0.04
Young adults (18-44)	22.7	24.7	23.7	23.0	24.0	23.2	24.3	23.5	24.6	22.5	
Middle-aged (45-64)	47.6	45.2	44.6	45.9	43.3	45.2	44.3	44.1	43.2	42.9	
Elderly (≥65)	29.8	30.1	31.8	31.1	32.8	31.7	31.4	32.4	32.2	34.7	
Female, %	67.2	65.9	67.0	64.8	64.9	65.8	65.2	66.6	65.2	63.9	0.227
Race, %											<0.001
White	60.2	60.3	63.8	62.7	63.8	62.0	64.9	65.0	63.0	63.9	
Black	16.2	17.5	17.5	17.6	17.6	18.7	17.4	17.5	19.5	19.5	
Hispanic	11.1	11.1	10.6	12.6	11.5	12.3	11.0	11.9	12.2	11.6	
Others	12.4	11.1	8.2	7.1	7.1	7.1	6.7	5.6	5.3	5.0	
CCI score, %											<0.001
0	41.0	39.0	39.8	40.7	41.6	41.2	39.1	36.9	39.3	37.4	
1	28.5	29.9	29.4	28.9	27.4	28.2	28.2	28.9	26.3	26.6	
2	15.6	15.0	14.5	13.4	14.7	13.8	14.3	14.4	14.0	14.8	
≥3	14.9	16.1	16.4	17.0	16.3	16.9	18.4	19.8	20.4	21.2	
Primary payer, %											<0.001
Medicare	39.4	39.5	39.7	40.5	40.4	38.6	38.8	40.3	39.1	39.7	
Medicaid	11.6	12.1	12.6	11.9	15.7	15.2	14.6	15.2	13.9	12.8	
Private insurance	41.4	41.3	39.0	39.3	38.3	41.8	41.8	40.1	41.0	41.8	
No insurance	7.6	7.1	8.8	8.3	5.6	4.4	4.9	4.5	5.9	5.7	
MHOI quartile, %											0.5381
1	30.4	30.6	33.1	32.3	31.5	33.2	30.4	31.2	29.8	31.7	
2	28.1	26.5	25.2	25.8	28.5	25.3	27.5	27.0	29.6	27.4	
3	24.1	24.7	24.4	24.5	23.2	23.3	25.2	25.0	24.1	24.1	
4	17.4	18.2	17.3	17.4	16.9	18.3	16.9	16.8	16.5	16.8	
Hospital bed size, %											<0.001
Small	12.7	12.5	13.6	14.8	20.8	20.9	21.3	22.9	23.1	24.9	
Medium	24.5	28.0	29.4	29.9	32.2	32.4	30.6	31.7	31.4	31.1	
Large	62.9	59.5	57.0	55.4	47.1	46.7	48.2	45.4	45.6	44.0	
Hospital region, %											0.9937
Northeast	16.2	19.0	17.7	17.0	17.5	16.5	17.4	17.6	16.9	16.6	
Midwest	25.4	22.5	23.5	23.4	24.2	23.8	25.8	25.6	24.7	24.6	
South	41.7	40.9	42.3	41.5	40.4	41.7	39.0	39.4	42.1	42.4	
West	16.7	17.6	16.5	18.2	17.9	18.0	17.8	17.4	16.3	16.4	
Location/teaching status of the hospital, %											<0.001
Rural	13.4	13.6	12.5	12.4	11.8	10.2	9.3	10.0	8.8	8.8	
Urban nonteaching	52.0	45.1	45.6	45.8	32.9	32.2	29.4	26.6	23.1	21.0	
Urban teaching	34.6	41.3	41.9	41.8	55.4	57.6	61.4	63.5	68.1	70.2	

When adjusted for age and gender for Joinpoint analysis, there was an average annual percentage change (AAPC) of 7.5% (CI = 5.5-9.4%, p < 0.01) in the incidence of MO among CDD hospitalizations from 2010 to 2019. Between 2010 and 2013, the annual percentage change (APC) was 12.4% (CI = 4.9-20.5%, p = 0.018). There was no significant trend in APC between 2013 and 2017 (APC = 2.1%, CI = -3.3 to 7.7%, p = 0.241). An APC of 11.3% (CI = 0.9-22.9%, p = 0.042) was noted between 2017 and 2019.

Biodemographic data

The biodemographic data of hospitalizations with CDD and MO are shown in Table 1. The mean age of hospitalizations showed a slight upward trend from 56.1 years in 2010 to 56.9 years in 2019 (p = 0.023). Also, there was a trend toward a reduced proportion of these hospitalizations in middle-aged patients and a trend toward an increase in the proportion of elderly patients (p = 0.04). The proportion of patients with a CCI score of 0 showed a downward trend over the study period (from 41% in 2010 to 37.4% in 2019) while that of patients with a CCI score ≥ 3 showed an upward trend (from 14.9% in 2010 to 21.2% in 2019), with p <0.001. There was an upward trend in the proportion of patients hospitalized in small and medium bed-size hospitals, and a marked increase in the proportion of patients hospitalized in urban teaching hospitals (from 34.6% in 2010 to 70.2% in 2019), both p < 0.001.

Outcomes

Table 2 shows the unadjusted outcomes of hospitalizations with CDD and MO.

**Table 2:** Unadjusted outcomes of hospitalizations with CDD and morbid obesity CDD: colonic diverticular disease; LOS: length of hospital stay; MHOI: median household income national quartile for patient's ZIP code; THC: total hospital charge.

Variable	2010	2011	2012	2013	2014	2015	2016	2017	2018	2019	p-value
Mortality rate, %											
Overall	0.7	0.6	0.6	0.5	0.6	0.8	0.5	0.3	0.4	0.4	0.313
Females	0.5	0.6	0.5	0.6	0.6	0.6	0.5	0.4	0.4	0.4	0.912
Males	1.1	0.5	0.6	0.3	0.5	1.0	0.4	0.3	0.3	0.3	0.135
Whites	1.0	0.7	0.6	0.5	0.5	0.9	0.7	0.4	0.3	0.4	0.07
Blacks	0.3	0.5	0.7	0.4	0.8	0.4	0.2	0.4	0.7	0.3	0.885
Hispanics	0	0.3	0.4	0.3	0.3	0.6	0	0	0.2	0.5	-
MHOI quartile 1	0.5	0.3	0.6	0	0.5	0.6	0.4	0.5	0.4	0.3	-
MHOI quartile 4	0.3	0.6	0.9	1.1	0.4	1.1	0.4	0.2	0.7	0.3	0.499
Mean LOS, days											
Overall	5.8	5.9	5.7	5.4	5.4	5.3	5.5	5.2	5.0	5.0	
Females	5.9	6.0	5.8	5.6	5.6	5.4	5.8	5.3	5.1	5.1	
Males	5.5	5.7	5.6	5.1	5.1	5.1	5.0	4.9	4.8	4.8	
Whites	5.9	6.0	5.8	5.4	5.5	5.5	5.6	5.2	4.9	5.1	
Blacks	5.8	6.0	6.0	5.6	5.3	5.1	5.4	5.0	5.2	5.0	
Hispanics	5.4	6.5	4.9	5.1	5.6	4.9	5.2	5.3	4.7	4.8	
MHOI quartile 1	5.5	5.9	5.8	5.3	5.5	5.4	5.8	5.0	4.8	5.1	
MHOI quartile 4	6.0	6.4	5.5	5.9	5.2	5.3	5.3	5.3	5.5	4.8	
Mean THC, US$											
Overall	46,923	53,480	50,964	49,613	51,123	52,670	55,842	54,608	54,103	56,057	
Females	46,827	53,345	50,067	49,812	51,798	52,323	57,735	55,732	54,544	56,082	
Males	47,119	53,743	52,775	49,243	49,858	53,338	52,345	52,374	53,279	56,011	
Whites	47,313	54,675	51,493	49,848	51,011	53,279	57,068	54,884	52,275	54,671	
Blacks	44,029	51,017	52,561	46,402	48,927	46,759	52,169	47,940	53,385	53,738	
Hispanics	56,355	67,438	51,936	58,185	63,164	62,413	59,420	63,801	61,698	68,683	
MHOI quartile 1	40,066	48,839	49,910	46,722	50,492	51,604	55,155	52,990	50,079	54,910	
MHOI quartile 4	52,465	61,193	54,726	57,974	55,691	59,261	62,010	62,465	63,654	59,072	

There was a 7.2% decline in mortality over the study period when adjusted for age, sex, and race (p = 0.011). Compared to young adults, the middle-aged and elderly had adjusted odds ratios (aOR) of 7.18 (95% CI = 2.2-23.3, p = 0.001) and 24.8 (95% CI = 7.9-77.9, p < 0.001), respectively, for mortality. There was no significant difference in adjusted mortality between males and females (p = 0.223). There was no significant difference in adjusted mortality among Blacks (p = 0.754) or Hispanics (p = 0.369) when compared to Whites. Among White hospitalizations, there was a 10% decline in mortality when adjusted for age and sex (p = 0.02). When Black hospitalizations were adjusted for age and sex, there was no significant trend in adjusted mortality (p = 0.737).

There was a 0.1 days reduction in adjusted overall LOS over the study period (p < 0.001). Middle-aged and elderly patients had increases in adjusted LOS of 0.36 (95% CI = 0.21-0.51, p < 0.001) and 0.76 (95% CI = 0.59-0.92, p < 0.001) when compared to young adults. LOS was 0.29 days higher in females compared to males (p < 0.001). There was a 0.11 days reduction in adjusted LOS among Whites and a 0.1 days reduction in adjusted LOS among Blacks over the study period (both p < 0.001).

There was a $668 increase in THC over the study period (p < 0.001). Middle-aged and elderly patients had increases in adjusted THC of $6,815 (p < 0.001) and $11,695 (p < 0.001) when compared to young adults. There was no significant difference in adjusted THC between males and females (p = 0.825).

## Discussion

We demonstrated that the incidence of MO in our cohort of patients increased over the study period. This is in keeping with concerns over the global obesity epidemic. From 1999 to 2020, the prevalence of obesity in the US increased from 30.5% to 41.9%. During the same time, the prevalence of MO rose from 4.7% to 9.2% [9].

Obesity has previously been shown to increase the likelihood of developing CDD. Positive associations were also found between weight gain and increased waist circumference/waist-to-hip ratio and the development of diverticular complications [13-15,25]. The exact biological mechanisms behind this association are still unclear, and the factors underlying the progression from diverticulosis to CDD remain poorly understood. Obesity is thought to be associated with connective tissue abnormalities such as altered cross-linking of elastin (an important extracellular matrix protein that provides resilience and elasticity to connective tissues and organs), which might explain the predisposition of obese individuals to the development of asymptomatic diverticulosis [4]. Obesity has also been associated with gut microbial dysbiosis, which might be necessary for the development of symptoms or complications such as acute diverticulitis and diverticular hemorrhage. Additionally, there is a link between obesity and chronic inflammation. Adipose tissue plays a key role in the regulation of immunity and the inflammatory response by secreting a number of cytokines known to participate in local and systemic inflammation. Therefore, obesity may enhance the subtle chronic inflammation present in patients with asymptomatic diverticulosis leading to muscular hypertrophy and enteric nerve remodeling with disordered motility, which leads to a vicious cycle resulting in eventual progression to CDD. Obesity may also precipitate the acute inflammatory process in diverticulitis and may influence diverticular bleeding through pathways that affect vascular integrity [4,17,25-27]. Other established risk factors for CDD include increasing age, low dietary fiber intake, and excessive consumption of red meat [15,27].

We found increased mean age and an increasing proportion of elderly patients in our study population. Similar findings have been demonstrated in other studies. In the US and many other developed nations, the prevalence of obesity in the elderly is increasingly in parallel to the general population-wide increase in obesity prevalence. This is likely due to lower physical activity and intrinsic age-related metabolic changes in the elderly [28]. Obesity in the elderly markedly reduces their quality of life and adds even more to the existing public healthcare burden associated with the elderly [29]. Obesity in the elderly is also associated with an increase in comorbidities [28], which (in addition to MO itself) may explain our findings of an increased proportion of patients with ≥3 CCI points.

Our study also showed that more of the admissions were catered for at small and medium-sized hospitals compared to large hospitals over the study period, but there was an increased proportion of patients managed at urban teaching hospitals. This could be explained by the increasing proportion of elderly patients and comorbidities leading to the need for care at a teaching hospital level. However, this may also reflect an increase in the complexity of CDD cases in relation to MO itself. Increased visceral fat and obesity have been associated with increased severity of the presentation of diverticulitis and an increase in complicated disease [13].

Despite this notable increase in MO prevalence and the probable increase in the severity and complications of CDD, we noted a decline in mortality and LOS over the study period. These improvements in overall outcomes are contrary to the findings of other retrospective reviews [30,31] that showed that patients with higher visceral/subcutaneous fat ratios and increasing body mass index were more likely to require emergency surgery, have longer hospital stays, and develop more complications, including superficial surgical site infection, deep incisional surgical site infection, organ space surgical site infection, wound disruption complications, ventilator dependence > 48 hours, acute renal failure, and return to the operating room. Neither study, however, demonstrated worse mortality outcomes. Our findings are likely explained by improved care in CDD admissions with modern medical advances (including improvement in laparoscopic and endoscopic techniques) and newer management guidelines [32]. Recent evidence supporting the treatment of uncomplicated acute diverticulitis without antibiotics has been associated with significantly shorter hospital stays [33,34].

Our study highlighted increased mortality, LOS, and THC in older patients compared to young adults. Old age has previously been shown to be an independent predictor of more severe CDD with worse outcomes, likely due to poorer functional status and the increased prevalence of coexisting comorbidities and frailty [35,36]. Our study also highlighted significantly higher LOS in females compared to males over the study period. While the reasons behind this are largely uncertain, prior studies have pointed toward worse CDD outcomes in females [37,38]. A large-scale nationwide retrospective cohort study conducted using CDC mortality data on over 55,000 US citizens who died from diverticulitis between 1999 and 2016 showed that females had disproportionately worse mortality outcomes than males. In the study, female patients had higher odds of dying at hospice facilities or nursing homes (odds ratio (OR) = 1.64; 95% CI = 1.55-1.73; p < 0.001) and lower odds of dying within the hospital compared with males (OR = 0.72; 95% CI = 0.69-0.75; p < 0.001) [37]. Our study did not demonstrate any difference in in-hospital mortality outcomes between males and females. Nonprocedural complications such as sepsis, obstructions, and chronic pelvic fistulae are more common in women and indicate that women often have more subtle presentations. These differences may reduce the likelihood of women undergoing invasive procedures when needed, resulting in even more severe disease and increased overall diverticulitis mortality as well as longer hospital stays [37,38].

Our study has some notable limitations. The NIS is an administrative database and is prone to the limitations associated with administrative databases. Firstly, ICD-9 and ICD-10 codes were utilized to document diagnoses that may not correspond completely to clinical and laboratory parameters. Thus, reported findings relied on an appropriate representation of administrative codes and accurate data collection. Secondly, the NIS contains data on admissions rather than individual patients. Patients with recurrent CDD may be documented as multiple admissions. Thirdly, the NIS lacks laboratory/radiologic data or data used to grade clinical disease severity; therefore, we could not stratify outcomes based on CDD severity. Lastly, we could not determine the effect of treatment modalities and medication adherence on outcomes. Nonetheless, this database provided a unique opportunity to study this cohort at a population level. Multiple centers are accounted for to ensure national representation.

## Conclusions

The incidence of MO increased among CDD hospitalizations over the study period. Mortality rate and LOS trended down from 2010 to 2019. These are likely due to improved care for patients hospitalized with complications of CDD. Outcomes were however worse in older patients compared to young adults, with increased LOS in females compared to males. These findings highlight the need for improved care in these subgroups. Further research on lifestyle and medical care for morbidly obese patients as well as amelioration of CDD complications in these patients is needed.
